# Effect of a prostaglandin F2α analogue on the cyclic corpus luteum during its refractory period in cows

**DOI:** 10.1186/1746-6148-8-220

**Published:** 2012-11-14

**Authors:** Beatrice Wenzinger, Ulrich Bleul

**Affiliations:** 1 Clinic for Reproductive Medicine, Vetsuisse-Faculty University of Zurich, Zurich, Switzerland

## Abstract

**Background:**

The goal of this study was to examine the response of the cyclic corpus luteum of cows to the administration of a prostaglandin F2α analogue (PGF) during the transition of refractoriness to responsiveness by investigating ultrasonographic changes in the corpus luteum and changes in plasma progesterone concentration in cows following the administration of PGF 3 and 5 days after ovulation.

**Results:**

All cows that responded to PGF given on day 5 ovulated on day 9. In the cows that did not respond on day 5, the luteal cross-sectional area stagnated after treatment, whereas the plasma progesterone concentration continued to increase. In the cows that received PGF on day 3 of the oestrous cycle, the luteal cross-sectional area and the plasma progesterone concentration continued to increase.

**Conclusions:**

The results of this study suggest that the transition of the bovine corpus luteum from refractoriness to responsiveness to PGF occurs during day 5 of the oestrous cycle because in 5 of 8 cows given PGF on day 5, early luteal regression was evident during the examination 24 hours after PGF administration.

## Background

Prostaglandin F_2α_ (PGF_2α_) and prostaglandin analogues (PGF) are luteolytic in cattle and other domestic species and usually induce oestrus when given during the luteal phase of the oestrous cycle. The interval between administration of the hormone and onset of oestrus ranges from 2 to 6 days [1]. Other than the dose and route of administration [2], follicular dynamics at the time of prostaglandin administration have a significant effect on this interval [1,3].

When prostaglandin is given in the first few days after ovulation, it does not induce luteolysis. The reason for this is that the cyclic bovine corpus luteum is refractory to prostaglandin for 5 to 7 days postovulation [4]. A single instillation of PGF_2α_ into the uterine horn ipsilateral to the corpus luteum 1 to 4 days post ovulation did not result in luteolysis, whereas instillation of PGF_2α_ on day 5 induced oestrus within 3 days [5]. When cows with a corpus luteum measuring at least 17 mm in diameter and a blood progesterone concentration of at least 0.8 ng/ml were given PGF_2α_, there was a continuous and significant decrease in the luteal cross-sectional area on ultrasonograms and the blood progesterone concentration within 24 hours [6]. In another study, the volume of the corpus luteum and the blood progesterone concentration of cyclic cows decreased significantly within 24 hours of PGF administration on day 10 of the cycle, whereas the hormone had no effect on the corpus luteum and blood progesterone concentration when given on day 4 [7].

Both large and small bovine luteal cells contain cytoplasmic electron-dense granules, and degranulation of these cells can be considered an early sign of luteolysis [8]. Following the administration of PGF, the number of granules decreases in large luteal cells but not in small luteal cells. Administration of PGF to cows before day 4 of the oestrus cycle does not result in degranulation of either cell type.

In contrast to cattle, mares given PGF on day 3 post ovulation underwent a decrease in the ultrasonographically detectable luteal tissue and a transient decrease in the blood progesterone concentration within 2 days. All the mares came into oestrus and ovulated on day 9 or 10 after PGF [9]. In jennies given PGF_2α_ on day 3 post ovulation, signs of oestrus were detected within 4 days and ovulation occurred 9 days after the hormone injection [10].

The goal of this study was to investigate ultrasonographic changes in the corpus luteum and changes in plasma progesterone concentration in cows following the administration of PGF 3 and 5 days after ovulation.

## Results

On day 3 after the first ovulation, eight of the 15 cows had a corpus luteum with a cavity and seven had a solid corpus luteum. Of the seven cows of group 1 (PGF on day 3), six ovulated between days 20 and 27 after the first ovulation. In the remaining cow, the corpus luteum regressed and could no longer be imaged on day 24, but no ovulation had occurred by day 32. Of the eight cows of group 2 (PGF on day 5), five responded and had a shortened cycle, followed by an ovulation on day 9 after the previous ovulation. Of the three remaining cows, which did not respond to PGF, two ovulated on day 21 and one on day 26. In groups 1 and 2, four and three cows, respectively, had a solid corpus luteum, and three and five cow, respectively, had a corpus luteum with a cavity. There were no double ovulations. The mean age of the cows did not differ between the two groups.

The development of luteal cross-sectional area and plasma progesterone concentration over time were monitored from day 2 to day 9 after the first ovulation, during which time a corpus luteum could be visualized ultrasonographically in all cows. After day 9 a corpus luteum could not be visualized ultrasonographically in all the cows of group 2 that responded to PGF. On days 2 to 9 there were no differences between the compact corpora lutea and corpora lutea with cavity with respect to cross-sectional area and plasma progesterone concentration.

**Figure 1:**
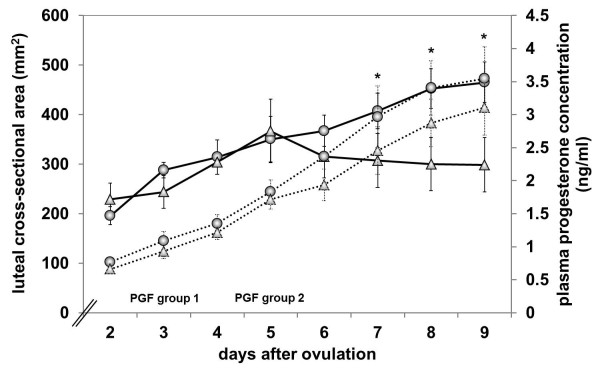
**Luteal cross**-**sectional area and plasma progesterone concentration in cows of group 1 and 2.** Luteal cross-sectional area in cows of group 1 (n = 7, ─○─) and in cows of group 2 that did not respond to PGF on day 5 (n = 3, ─Δ─) and plasma progesterone concentration in cows of group 1 (n = 7, · ○ ·) and in cows of group 2 that did not respond (n = 3, · Δ ·) from day 2 to day 9 after PGF. * Significant difference between the two groups in their luteal cross-sectional area.

**Figure 2:**
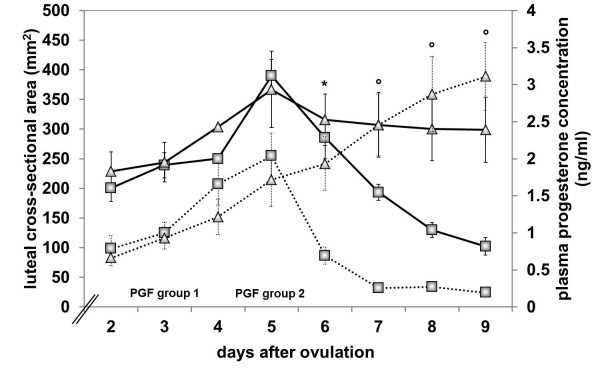
**Luteal cross**-**sectional area and plasma progesterone concentration in cows of group 2.** Luteal cross-sectional area in cows of group 2 that responded to PGF administered on day 5 of the oestrous cycle (n = 5, ─□─) and in cows of group 2 that did not respond (n = 3, ─Δ─) and plasma progesterone concentration in responding (n = 7, · □ ·) and non-responding cows of group 2 (n = 3, · Δ ·) from day 2 to day 9 after PGF. * Significant difference between the two groups in their plasma progesterone concentration. °Significant difference between the two groups in their luteal cross-sectional area and plasma progesterone concentration.

Based on the luteal cross-sectional area, the corpora lutea of the cows in group 1 increased progressively in size from day 2 (a = 195.97 ± 18.43 mm^2^) to day 9 (a = 465.38 ± 40.88 mm^2^, *P* ≤ 0.0001), which was accompanied by a progressive increase in plasma progesterone concentration (P4 = 0.71 ± 0.09 ng/ml on day 2 to P4 = 2.13 ± 0.48 ng/ml on day 9, *P* ≤ 0.0001, Figure 1). In cows of group 2 that responded clinically to PGF (n=5), the increase in luteal cross-sectional area and plasma progesterone concentration until day 5 and the decrease in both variables thereafter were largely parallel (Figure 2). The luteal cross-sectional area increased significantly from 200.34 ± 22.56 mm^2^ on day 2 to a maximum of 390.08 ± 26.92 mm^2^ on day 5, the day of PGF application (*P* ≤ 0.0001), after which it decreased to 298.81 ± 54.86 mm^2^ on day 9 (*P* ≤ 0.0001). Values for plasma progesterone concentration increased significantly from day 2 (P4 = 0.79 ± 0.18 ng/ml) to day 5 (P4 = 2.04 ± 0.31 ng/ml, *P* ≤ 0.0001) and decreased thereafter until day 9 (P4 = 0.19 ± 0.04 ng/ml, *P* ≤ 0.0001). In the cows of group 2 that did not respond to PGF (n=3), the curves of the luteal cross-sectional area and plasma progesterone concentration differed significantly (*P* ≤ 0.0001); there was a progressive increase in plasma progesterone concentration, whereas the luteal cross-sectional area had a plateau after day 5 (Figures 1 and 2). Progesterone concentration in these cows increased from 0.66 ± 0.11 ng/ml on day 2 to 3.11 ± 0.46 ng/ml on day 9 (*P* = 0.0001). The values of luteal cross-sectional area increased from 229.11 ± 32.38 mm^2^ on day 2 to 369.14 ± 69.23 mm^2^ on day 5 (*P* ≤ 0.0001), fell thereafter to 315.79 ± 43.6 mm^2^ on day 6 (*P* ≤ 0.0001) and decreased only slightly to day 9 (298.81 ± 54.86 mm^2^). Values of luteal cross-sectional area in cows of group 2 that did not clinically respond to PGF were significantly lower than values in cows of group 1 after day 7 (*P* = 0.0350, Figure 1). Compared to cows of group 2 which responded to PGF, values of luteal cross-sectional area of the cows in group 2 that did not respond to PGF were significantly higher after day 7 (*P* = 0.0405, Figure 2). Plasma progesterone concentration differed significantly between the cows of group 2 that responded to PGF and those that did not already after day 6 (*P* = 0.0010, Figure 2).

## Discussion

Five of eight cows (62.5%), that were treated with PGF on day 5 after ovulation, had a shortened cycle. In these cows, a significant decrease in luteal cross-sectional area as well as in plasma progesterone concentration occurred within one day after PGF treatment, confirming similar findings by Levy and others [4] and Repasi and others [6]. All five cows of the present study that responded to PGF given on day 5 ovulated on day 9. The remaining three cows (37.5%) had a progressive increase in plasma progesterone after the application of PGF, but the luteal cross-sectional area remained constant until day 9.

The likelihood that PGF causes luteolysis in cows increases with increasing plasma progesterone concentration [11]. Although the differences in the present study were not significant, there was a trend for higher plasma progesterone concentration in cows of group 2 that responded to PGF than in cows that did not respond. Likewise, when PGF was given on day 5, the responding cows had numerically higher plasma progesterone concentrations than the non-responding cows. Because ultrasonographic examinations were only carried out once a day, the maximum difference between cycle stages among individual cows was 24 hours. This could explain some of the variation in the refractoriness of the corpus luteum in the cows of group 2.

The courses of the luteal cross-sectional area and plasma progesterone concentration from day 2 to day 9 were largely parallel in cows of group 1 and those cows of group 2 that responded to PGF. These findings are in agreement with the findings from Lüttgenau and others [12], that plasma progesterone concentrations in the mid-luteal phase are dependent on luteal size. In contrast, in the cows of group 2 that did not respond, the luteal cross-sectional area reached the maximum on day 5 and remained at that level until day 9, whereas the plasma progesterone concentration progressively increased until day 9. This suggests that the application of PGF during the presumed transition of the bovine corpus luteum from refractoriness to responsiveness on day 5 affected the growth of the corpus luteum, but not synthesis and secretion of progesterone. Similar observations were made in mares, in which the luteal cross-sectional area decreased continually until day 9 following PGF on day 3, whereas the plasma progesterone concentration decreased within one day, then temporarily increased during the next two days before it decreased again to below 1.0 ng/ml, when the mares started to show signs of heat [9].

As expected, administration of PGF on day 3 did not shorten the oestrous cycle. However there might be a possible correlation between the injection of PGF on day 3 and the particular delay in the following ovulations. The application of PGF did not alter the normal increases in luteal cross-sectional area or the plasma progesterone concentration. In agreement with the aforementioned studies, treatment of cows in our study with a luteolytic dose of PGF on day 3 of the oestrous cycle did not result in changes of the ultrasonographic appearance of the corpus luteum, and had no effect on the progressive increase in plasma progesterone. The increase in plasma progesterone concentration in cows of group 1 reflected the typical progesterone profile established through daily blood sampling during a natural oestrous cycle [13]. Rowson and co-workers [5] were the first to show that a single injection of PGF2α on days 1 to 4 of the bovine oestrous cycle (day of ovulation = day 0) does not induce a new cycle. In a study on the effect of PGF2α on degranulation of bovine luteal cells, no structural or functional changes were detected in luteal cells 8 days after a prostaglandin injection on day 4, as assessed by electron microscopy and measurement of luteal progesterone concentrations, respectively [8]. In another study the administration of PGF2α in cows on day 4 of the oestrous cycle did not affect the increasing plasma progesterone concentration [4]. In a study that examined the relationship between cycle stage and response to PGF in cattle, 43.0%, 83.6% and 100% of cows treated on days 5 to 7, 8 to 11 and 12 to 15, respectively, came into heat [14]. Lane and others [15] reported an immediate regression of the corpus luteum, if PGF is administered from day 6 of the oestrous cycle.

## Conclusions

Based on the results of the present study, it can be assumed that the transition of the bovine corpus luteum from refractoriness to responsiveness occurs on day 5 of the oestrous cycle because in 5 of 8 cows given PGF on day 5, early luteal regression was evident during the examination 24 h after PGF. It is assumed that the type of response of the corpus luteum to PGF depends on the exact age of the corpus luteum at the time of treatment. Therefore, for a more precise determination of the period of refractoriness of the bovine corpus luteum to PGF, more frequent examinations on the day of ovulation are required for a more precise diagnosis of ovulation. Knowledge of the exact time of ovulation should facilitate the determination of the period of refractoriness.

## Methods

The trial protocol was ethically approved by the Animal Care Committee of the Canton of Zurich, Switzerland.

Fifteen cows (13 Swiss Braunvieh and two Simmental) ranging in age from four to 15 years were used. They all originated from a small farm where they were kept in a tie-stall barn during winter season and on pasture during summer. There was twice a day milking and the feed consisted of grass, hay, grass silage and maize silage. For performing the study the cows were brought to the animal hospital. There they were tied on straw, fed hay and grass silage and were also milked twice a day. The cows used to conduct this study were low-yielding cows with an average milk yield of 15 kg a day. The cows had to be between 60 and 100 days postpartum and in dioestrus. A thorough ultrasonographic examination of the uterus and ovaries of all cows revealed no abnormalities. Only cows with at least one corpus luteum with a minimum cross-sectional area of 22 x 20 to 25 mm were included in the study, the others were examined in an interval of 2 to 3 days and included in the study to a later point of time; a corpus luteum of this size was defined as producing progesterone [16].

The study was conducted at the animal hospital in Zurich, Switzerland (latitude 47.4°, longitude 8.55°, 420 metres above sea level). All cows were treated with 15 mg of the synthetic PGF_2α_ analogue Luprostiol (Prosolvin®, Veterinaria, Zurich, Switzerland), administered intramuscularly into the semitendinous muscle. Thereafter the cows were examined ultrasonographically every morning to identify and measure follicles and corpora lutea using a real-time, B-mode, ultrasound scanner with an 8.0 MHz linear-array transducer and built-in electronic calipers (Aquila, Pie Medical, Maastricht, The Netherlands). The day on which the dominant follicle was not visible for the first time was defined as the day of ovulation (day 0). After ovulation, the cows were allocated alternately to group 1 or group 2 and received a second treatment of prostaglandin on day 3 (n=7) or day 5 (n=8), respectively. Maximum width and height of the corpus luteum and of cavities in the luteal tissue were measured and the cross-sectional area of the luteal tissue was calculated. The area of a solid corpus luteum without a cavity was calculated using the formula: area = a = w_Cl_ × h_Cl_ × *π*/4, and the area of a corpus luteum with a cavity was calculated using the formula: area = w_Cl_ × h_Cl_ × *π*/4 − w_C_ × h_C_ × *π*/4 (w, width; h, height; Cl, corpus luteum; C, cavity) [17,18]. Blood samples for progesterone measurement were collected daily from the tail vein into an evacuated lithium-heparin tube. The blood was centrifuged for 10 min at 3,000 rpm and the plasma was removed and stored at −20°C. The plasma progesterone concentration was measured at the Department for Analytical Chemistry and Endocrinology, University of Veterinary Medicine, Hannover, Germany, using a competitive chemiluminescence-immunoassay with an analytical assay sensitivity of 0.4 pg [19]. The intra- and inter-assay precision for the enzyme-immunoassay was 5.2 and 9.5 % for low references and 6.2 and 5.4 % for high references.

Daily ultrasonographic examinations and blood sampling for progesterone measurement were continued until ovulation had occurred after the second prostaglandin injection, which varied from 12 to 32 days. The data obtained from day 2 to day 9 after ovulation induced by the first prostaglandin injection were used for analysis with StatView 5.0 (SAS Institute, Wangen, Switzerland). The unpaired student’s t-test was used to examine if the luteal area and the progesterone concentration were different between compact corpora lutea and corpora lutea with cavity on days 3, 5 and 7. The same test was used to compare the luteal area and the progesterone concentration between the cows treated on day 3 or 5. Repeated measures of ANOVA were conducted to compare the development of the luteal cross-sectional area and progesterone concentrations between cows that were treated on day 3 with PGF and cows that were treated on day 5 but exhibited no luteolysis and between cows that were treated on day 5 with PGF that exhibited a luteolysis and cows that did not. Results were presented as mean ± SEM. A value of *P* ≤ 0.05 was considered significant.

## Competing interests

Both authors declare that they have no competing interest.

## Authors’ contributions

UB initiated and planned the study. BW carried out the ultrasonographic examinations and the sampling of blood. BW also performed the statistical analysis and drafted the manuscript. Both authors read and approved the final manuscript.
